# Supplementary material for: Extracellular Calcium Ion Concentration Regulates Chondrocyte Elastic Modulus and Adhesion Behavior
Source: Int J Mol Sci. 2021 Sep 17;22(18):10034. doi: 10.3390/ijms221810034 (PMC8468569; doi:10.3390/ijms221810034)
Supplement: Supplementary file 1 [file ijms-22-10034-s001.zip › ijms-1323294-supplementary.pdf]

Supporting Information

Extracellular Calcium ion Concentration Regulates Chondrocyte Elastic Modulus and Adhesion Behavior

Xingyu Shen [1], Liqiu Hu [1], Zhen Li [2], Liyun Wang [3], Xiangchao Pang [4], Chun-Yi Wen [5] and Bin Tang [1,*]

[1] Department of Biomedical Engineering, Southern University of Science and Technology, Shenzhen, 518055, China; 11749079@mail.sustc.edu.cn (S.X.); 11930748@mail.sustech.edu.cn (H.L.)

[2] School of Chemistry and Environmental Engineering, Jiangsu University of Technology, Changzhou 213001, China; lizhen198458@163.com

[3] Center for Biomechanical Research, Department of Mechanical Engineering, University of Delaware, Newark, DE, 19716, USA; lywang@udel.edu

[4] College of Materials Science and Engineering, Central South University of Forestry and Technology, Changsha, 410004, China; T20172373@csuft.edu.cn

[5] Department of Biomedical Engineering, The Hong Kong Polytechnic University, Hong Kong, 999077, China; chunyi.wen@polyu.edu.hk

* Correspondence: tangb@sustech.edu.cn

Table

Table S1. The precise values of the results.

Ca^{2+} concentration / mM	1.75	2.25	2.75	3.25	3.75	Control
Young's modulus / KPa	1202 ± 250	1521 ± 75	1644 ± 396	1402 ± 211	1510 ± 155	
F_{max} / nN	1103± 439	1298 777	1632 ± 724	1295 ± 526	1208 ± 633	130 ± 90
W_d / 10^{-15}J	1.3 ± 0.8	1.8 ± 0.6	2.3 ± 0.9	1.9 ± 0.9	1.6 ± 0.9	0.1 ± 0.1
F_{max} / nN (CWHM-12)	308 ± 172	300 ± 200	410 ± 217	304 ± 149	381 ± 203	
W_d / 10^{-16}J (CWHM-12)	3.6 ± 2.8	4.4 ± 4.2	3.8 ± 2.8	3.8 ± 2.7	4.6 ± 3.1	
$(F_{max}$ - F_{max} (CWHM-12))/ F_{max}	72%	77%	75%	76%	68%	
(Wd - Wd(CWHM-12))/Wd	72%	76%	83%	80%	71%	

Table S2. Primers for qPCR.

Gene	Sequences (5′–3′)
	Forward primer: GCTCCTCCTGAGCGCAAGTAC

Gene	Sequences (5′–3′)
β-actin	Reverse primer: GGACTCGTCATACTCCTGCTTGC
col2a1	Forward primer: CGCCGCTGTCCTTCGGTGTC<br>Reverse primer: AGGGCTCCGGCTTCCACACAT
acan	Forward primer: TGGGAACCAGCCTATACCCCAG<br>Reverse primer: CAGTTGCAGAAGGGCCTTCTGTAC
myosin	Forward primer: CAGCAAGCTGCCGATAAGTAT<br>Reverse primer: CTTGTCGGAAGGCACCCAT
integrin beta 1	Forward primer: CCTACTTCTGCACGATGTGATG<br>Reverse primer: CCTTTGCTACGGTTGGTTACATT
integrin beta 3	Forward primer: GTGACCTGAAGGAGAATCTGC<br>Reverse primer: CCGGAGTGCAATCCTCTGG
collagen Ⅵ	Forward primer: ACAGTGACGAGGTGGAGATCA<br>Reverse primer: GATAGCGCAGTCGGTGTAGG
Collagen Ⅰ	Forward primer: CGCCGCTGTCCTTCGGTGTC<br>Reverse primer: AGGGCTCCGGCTTCCACCACAT

Figure

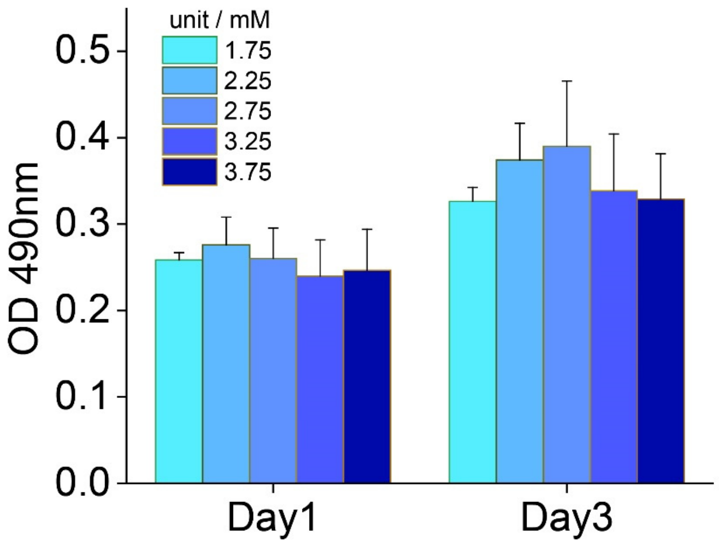

Figure S1. MTT assay shows the proliferation of chondrocytes at day 1 and day 3. No statistic difference in each groups.

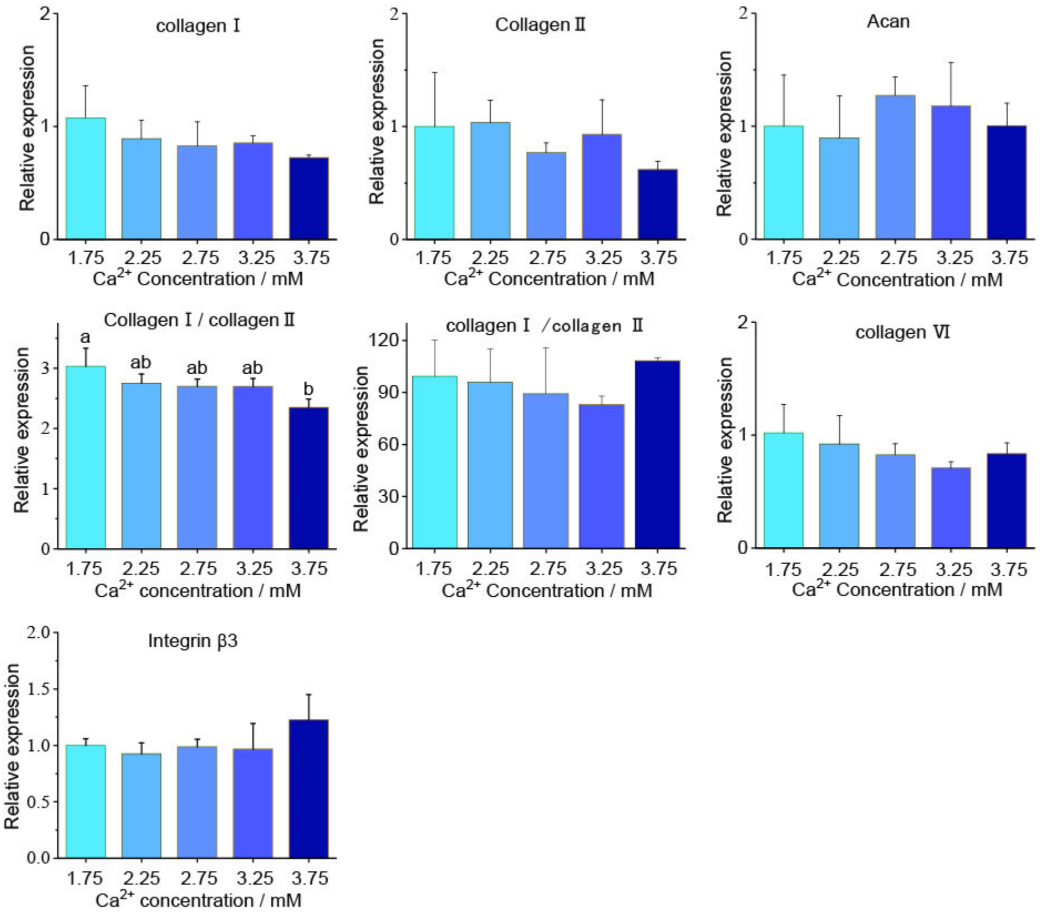

Figure S2. The expression of *collagen Ⅱ, collagen Ⅰ, collagen Ⅵ, integrinβ3, and aggrecan* has no significant difference.

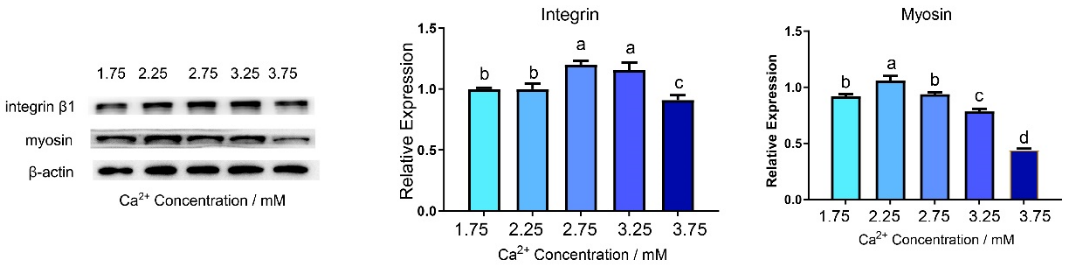

Figure S3. Western blot shows that the trend of *myosin Ⅱ* and *integrin β1* expression level is similar to that of qPCR and immunofluorescence results.

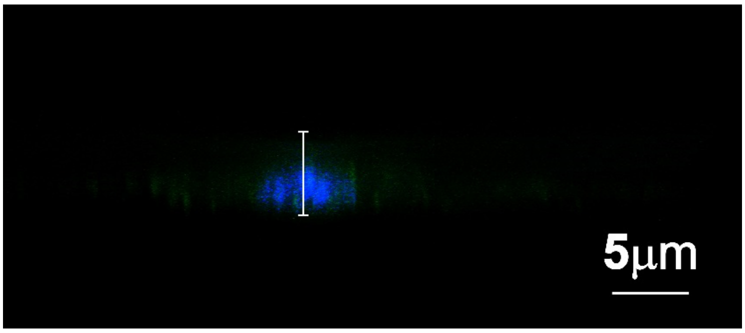

Figure S4. The height of chondrocytes was about 6 μm measured by confocal laser scanning microscope. Blue: DAPI; green: phalloidin.
